# Post-Burn Quality of Life, Post-Traumatic Stress Disorder (PTSD), and Insomnia: A Six-Month Follow-Up Study

**DOI:** 10.7759/cureus.97944

**Published:** 2025-11-27

**Authors:** Ilias Tsiampouris, Maria Polikandrioti, Ioannis Koutelekos, Irene E Gamatsi, Christos Gakis, Panagiota Makrygianni, Andriana Maggita, Georgios Vasilopoulos

**Affiliations:** 1 Department of Nursing, University of West Attica, Athens, GRC; 2 Department of Plastic and Reconstructive Surgery, Melanoma Reference Center, General Hospital of Athens “G. Gennimatas”, Athens, GRC; 3 Department of Plastic and Reconstructive Surgery, KAT General Hospital, Athens, GRC

**Keywords:** burn disease, burn injury, insomnia, post-traumatic stress disorder, quality of life

## Abstract

Introduction: Burn injuries significantly impact patients' quality of life (QoL), often leading to insomnia and post-traumatic stress disorder (PTSD). The aim of the present research was to explore QoL, PTSD, and insomnia in patients with burn injuries three and six months after hospital discharge, as well as the associated factors with QoL.

Material and methods: The present study enrolled adults with burn injuries in any area of the body who were measured at three and six months after the injury. The research tools were Burn Specific Health Scale-Brief, Abbreviated Burn Severity Index, Athens Insomnia Scale, and Impact of Event Scale-Revised, which also included patients’ demographic and clinical data as well as reports from patients. Statistical significance was set at p<0.05, and analyses were conducted using IBM Corp. Released 2018. IBM SPSS Statistics for Windows, Version 27.0. Armonk, NY: IBM Corp.

Results: Data from 85 patients showed mild-to-moderate PTSD symptoms (IES-R mean 2.75 at three months, and 2.59 at six months; p=0.246). From three months to six months, measurement of QoL significantly improved (p<0.001), while insomnia symptoms decreased (p<0.001). PTSD and insomnia were negatively correlated with QoL at three and six months (p<0.001) and positively associated with each other at both measurements (p<0.001). Greater burn extent predicted worse QoL in Hand function and overall score (p=0.006, p<0.001). PTSD symptoms were linked to multiple QoL domains (e.g., affect p<0.001, sexuality p=0.001), while insomnia predicted worse simple abilities (p<0.001).

Conclusion: This study highlights the complex interplay between QoL, insomnia, and PTSD in patients with burn injuries. Further research with larger, more representative samples and extended follow-up is essential to develop effective interventions and improve patient outcomes.

## Introduction

Burn injuries constitute severe trauma affecting not only physical integrity but also patients’ mental health and quality of life (QoL). Burn recovery is a multidimensional process involving the management of physical, psychological, and social challenges. Among the key factors influencing burn recovery, QoL, insomnia, and post-traumatic stress disorder (PTSD) have received increasing attention in recent research [1,2].

According to the World Health Organization (WHO), QoL refers to an individual's perception of their position in life, within the context of their culture and value system, and in relation to their goals, expectations, standards, and concerns. In individuals with burn injuries, QoL is often substantially impaired due to persistent pain, functional limitations, aesthetic alterations, and social repercussions. Patients with severe burn injuries frequently encounter obstacles in returning to social roles and employment, further compromising their QoL [2-4].

Sleep disturbances, particularly insomnia, are commonly reported by patients with burn injuries. Factors such as pain, anxiety, and depression frequently disrupt sleep patterns, contributing to chronic insomnia and further deteriorating both physical and mental health. In addition, chronic insomnia may increase the risk of developing psychiatric disorders, including depression and anxiety [2,5].

Post-traumatic stress disorder (PTSD) is a psychiatric condition that may develop after exposure to or witnessing a traumatic event, such as a severe burn injury. Symptoms include flashbacks, nightmares, avoidance of trauma-related stimuli, negative mood alterations, and hyperarousal. PTSD is prevalent among burn survivors and significantly affects their psychological well-being and capacity to reintegrate into daily life [6,7].

All aforementioned factors exert a negative influence on QoL and constitute an impediment to recovery. Interestingly, the relationship between QoL, insomnia, and PTSD in patients with burn injuries is complex and bidirectional. For instance, insomnia may exacerbate PTSD symptoms, while PTSD can further disrupt sleep. Both conditions have been associated with diminished QoL, creating a vicious cycle that impedes long-term recovery. Understanding these dynamics is essential for developing effective interventions with the ultimate goal of improving the overall well-being of burn survivors [8,9].

Despite the acknowledged importance of QoL, insomnia, and PTSD as critical factors in burn survivors, the literature remains limited in exploring their interrelations, particularly the impact of insomnia and PTSD on QoL outcomes. However, longitudinal studies have commonly employed three- and six-month follow-up intervals to track recovery trajectories. For example, a 12-month prospective physiotherapy outcomes study conducted assessments at 1, 3, 6, and 12 months, demonstrating that most functional outcomes improved close to baseline by six months post-injury. Similarly, an exploratory multicenter study assessed HRQL at three, six, 12, and 18 months post-burn, revealing that by 18 months, patients often returned to population-norm levels of HRQL. Furthermore, the absence of standardised assessment instruments and evidence-based intervention protocols further impedes the provision of comprehensive and effective care [10,11].

The present study aimed to examine QoL, PTSD, and insomnia in patients with burn injuries at three- and six-month post-discharge, and secondarily to identify factors associated with QoL outcomes.

## Materials and methods

Study design, setting, and period

This study was conducted from 2023 to 2025 in the outpatient clinics of two public hospitals in Attica, Greece. Adult burn survivors with burn injuries affecting any area of the body who visited the clinics for regular follow-up were recruited using a convenience sampling method.

Inclusion and exclusion criteria

Eligible participants met the following criteria: (i) ability to understand and speak the Greek language; (ii) cognitive capacity to comprehend the study’s purpose and procedures; (iii) age >18 years old; (iv) deep partial-thickness burn injury, involving damage through most of the dermis, often with severe pain, blistering, and a moist wound surface, affecting >10% of total body surface area (TBSA), and/or full-thickness burn injury, destroying all skin layers, leaving the area dry, leathery, and without sensation, and requiring surgical intervention, affecting >5% of TBSA, involving any area of the body; (v) any type of burn injury, regardless of cause; (vi) hospitalization in a Burn Unit or Plastic Surgery Department for >15 days. Exclusion criteria included a diagnosed psychiatric or neurological disorder that significantly limited physical functioning or communication.

Data collection procedure

Data were collected by the researcher through face-to-face interviews, during which the research questionnaires were administered and completed. For each patient, the interview took place in a private office setting after the follow-up assessment. Completing all questionnaires required approximately 20 minutes.

Institutional review board statement

The present study was approved by the Research Committee of the public hospitals (decision 5th/15-05-2023 for Georgios Gennimatas Hospital and 6th/05-04-2023 for KAT Hospital). Ethical approval was obtained from the Ethics Committee of the University of West Attica, under the supervision of the Department of Nursing (Approval Code: 1/12-01-2023). Patients who met the entry criteria were informed by the researcher about the purposes of this study. Written informed consent was obtained for all patients being interviewed. Data collection guaranteed anonymity and confidentiality. All subjects had been informed of their rights to refuse or discontinue participation in the study, according to the ethical standards of the Declaration of Helsinki (1989) of the World Medical Association.

Informed consent statement

Informed consent was obtained from all subjects involved in the study.

Research instruments

The study collected data across three domains: (i) Demographic characteristics (e.g., gender, age, ethnicity, educational level); (ii) burn-related variables (e.g., burn type, extent and depth of injury); (iii) clinical variables (e.g., duration of hospitalization, type of treatment, skin grafts, complications, and clinical outcome). Outcomes were classified as complete healing, discharge under monitoring, scheduled readmission, or death.

Measurement of quality of life

The Burn Specific Health Scale-Brief (BSHS-B) was used to assess QoL. This 40-item questionnaire includes nine domains: heat sensitivity, hand function, treatment regimen, work capacity, affect (impact of the injury on daily life), sexuality, interpersonal relationships, simple abilities, and body image. Responses are rated on a five-point Likert scale, with higher scores indicating better QoL. According to the original validation study, all domains demonstrated Cronbach’s α coefficients ranging from 0.75 to 0.93, while in the Greek adaptation of the instrument, the overall Cronbach’s α was 0.954, indicating excellent internal consistency [12]. The Burn Specific Health Scale-Brief (BSHS-B) was used with permission from the copyright holders. 

Measurement of burn severity

The Abbreviated Burn Severity Index (ABSI) was used to assess burn injury severity, incorporating five variables: age, gender, inhalation injury, presence of full-thickness burn injuries, and TBSA affected. ABSI scores are categorized into six groups (2-3, 4-5, 6-7, 8-9, 10-11, and ≥12), with lower scores indicating a higher chance of survival. As the ABSI is primarily a prognostic scoring system rather than a psychometric tool, studies typically do not report Cronbach’s alpha (α) for this measure. A recent meta-analysis published in PLOS ONE (2025) confirmed the ABSI’s role as a reliable prognostic indicator in severely burned patients, demonstrating that higher ABSI scores are strongly associated with increased mortality risk [13-15].

Measurement of insomnia

The Athens Insomnia Scale (AIS) was used to evaluate the presence of insomnia. The AIS includes eight items, five assessing nighttime sleep and three assessing daytime functioning. Each item is scored on a four-point Likert scale (0 = not at all, 3 = very much). A total score of ≥6 indicates the presence of insomnia. The original validation study of the AIS, developed by Greek researchers and standardized on Greek patients, reported a Cronbach’s α of 0.89, indicating high internal consistency. Since its introduction, the AIS has been officially translated and culturally adapted into multiple languages, demonstrating its applicability across diverse populations [16].

Measurement of post-traumatic stress disorder

Post-traumatic stress symptoms were assessed using the Impact of Event Scale-Revised-Greek version (IES-R-Gr). This 22-item instrument includes three subscales: intrusion (eight items), avoidance (eight items), and hyperarousal (six items). Each item is rated on a five-point Likert scale (0 = not at all, 4 = extremely), yielding a total score between 0 and 88. PTSD symptom severity is classified as follows: 0-8 (subclinical), 9-25 (mild), 26-43 (moderate), and ≥44 (severe). A score of ≥26 indicates clinically relevant post-traumatic stress symptoms. According to the original validation study of the IES-R, Cronbach’s α coefficients were 0.87 for intrusion, 0.84 for avoidance, and 0.79 for hyperarousal, while in the Greek adaptation, values were 0.72, 0.77, and 0.845, respectively. In both cases, no total Cronbach’s α was reported [17].

Statistical analysis

Quantitative variables were tested for normality using the Kolmogorov-Smirnov criterion and were expressed as mean values (standard deviation) and as median (interquartile range), while categorical variables were expressed as absolute and relative frequencies. The Wilcoxon signed-rank test was used for comparing patients’ scores in the IES-R, BSHS-B, and AIS scales between the two measurements. The McNemar test was used to compare the percentage of insomnia between the two measurements. Spearman correlation coefficients (rho) were used to explore the association among the under-study scales. The coefficient is considered very high when it is above 0.9, high when it is 0.7-0.9, moderate when it is 0.5-0.7, low when it is 0.3-0.5, and very low when it is below 0.3. Multiple linear regression analysis was used with the dependent variables, the IES-R and BSHS-B scales at each measurement in a stepwise method (p for entry 0.05, p for removal 0.10). All patients’ characteristics were entered in the analyses. When BSHS-B scales were the dependent variables, IES-R and having insomnia were also included in the analyses. Log transformations of the dependent variables were used for the multiple linear regression analyses. Logistic regression analysis in a stepwise method (p for entry 0.05, p for removal 0.10) was used in order to find independent factors. Adjusted odds ratios (OR) with 95% confidence intervals (95% CI) were computed from the results of the logistic regression analyses. All reported p-values are two-tailed. Statistical significance was set at p<0.05, and analyses were conducted using IBM Corp. Released 2018. IBM SPSS Statistics for Windows, Version 27.0. Armonk, NY: IBM Corp.

## Results

Data were collected and analyzed from 85 patients. Their demographic and clinical characteristics are presented in Tables 1, 2, respectively. The mean age was 54.4±18 years, and the mean duration of hospitalization was 37.5±28.1 days. The majority of patients were males (60%), of Greek ethnicity (85.9%), and residing in Attica (74.1%). Regarding clinical characteristics, most patients received surgical treatment (87.1%), 71.8% had achieved complete healing, and one patient had died. Thermal burn injuries were observed in 88.2% of cases, and deep partial-thickness burn injuries in 91.8%. The mean burn extent was 22.1%±13%, and the mean ABSI score was 6.7±2.1.

**Table 1:** Distribution of the sample according to demographic characteristics (n=85).

Demographic characteristics		Mean ± SD
Gender	Males	51.0±60
	Females	34.0±40
Age (years), mean (SD)		54.4±18
Nationality	Greek	73.0±85.9
	Other	12.0±14.1
Place of residence	Attica	63.0±74.1
	Out of Attica	20.0±23.5
	Out of Greece	2.0±2.4
Educational level	Primary school	6.0±7.1
	Secondary school	11.0±12.9
	High school	41.0±48.2
	University	19.0±22.4
	MSc	8.0±9.4
Type of family	Nuclear family	53.0±62.4
	Extended family	4.0±4.7
	Single-parent	1.0±1.2
	Alone	27.0±31.8

**Table 2:** Distribution of the sample according to clinical characteristics (n=85).

Clinical characteristics		Mean ± SD
Duration of hospitalization (days), mean (SD)		37.5±28.1
Comorbidities		23.0±27.1
Treatment of burn	Conservative	11.0±12.9
	Surgery	74.0±87.1
Escharotomy		19.0±22.4
Autograft transplantation		33.0±38.8
Complications		42.0±49.4
Presence of inhalation injury		11.0±12.9
Outcome	Complete healing	61.0±71.8
	Exit under surveillance	21.0±24.7
	Exit and scheduled readmission	2.0±2.4
	Death	1.0±1.2
Type of burn	Thermal	75.0±88.2
	Chemical	6.0±7.1
	Electric	3.0±3.5
	Radiation	0.0±0
	Friction	1.0±1.2
Burn depth	Superficial	42.0±49.4
	Superficial partial	56.0±65.9
	Deep partial	78.0±91.8
	Full thickness	35.0±41.2
	Mixed	68.0±80.0
Extent of burn (%), mean (SD)		22.1±13
ABSI Score, mean (SD)		6.7±2.1
Probability of surviving	>99%	6.0±7.1
	98%	16.0±18.8
	80-90%	39.0±45.9
	50-70%	16.0±18.8
	20-40%	5.0±5.9
	<10	3.0±3.5

Table 3 presents scores on the IES-R, BSHS-B, and AIS scales at both measurement points. At three months post-discharge, the mean total IES-R score was 2.75 (SD = 1.55), while at six months it was 2.59 (SD = 1.47), with no statistically significant change (p = 0.246). Higher IES-R scores indicate more severe PTSD symptoms, with values above 1.5-1.6 considered clinically significant. Total BSHS-B score increased significantly from 102.15 (SD = 25.79) to 122.21 (SD = 24.82) (p < 0.001), indicating a notable improvement in QoL. AIS scores decreased from 8.32 (SD = 4.69) to 5.92 (SD = 3.9) (p < 0.001), suggesting reduced insomnia symptoms. Scores ≥6 denote the presence of insomnia, and scores ≥10 indicate clinically significant insomnia. Overall, patients showed mild-to-moderate PTSD symptoms, improved QoL, and reduced insomnia levels at follow-up. A more detailed analysis revealed a significant reduction in IES-R avoidance subscale scores at six months, from 0.78 (SD = 0.54) to 0.65 (SD = 0.56) (p = 0.038), while changes in the intrusion and hyperarousal subscales were not statistically significant. Significant QoL improvements were noted at six months in the following domains: Simple abilities from 1.12 (SD = 1.15) to 3.05 (SD = 0.95) (p < 0.001); hand function from 2.5 (SD = 1.34) to 3.74 (SD = 0.47) (p < 0.001); treatment regimens from 2.17 (SD = 1.06) to 2.95 (SD = 1.05) (p < 0.001); work from 1.56 (SD = 1.23) to 2.44 (SD = 1.02) (p < 0.001); interpersonal relationships from 3.73 (SD = 0.35) to 3.57 (SD = 0.56) (p = 0.001). The mean increase in total BSHS-B score was 20.06 (SD = 21.55); p < 0.001. The AIS score decreased significantly by 2.4 points (SD = 5.21); p < 0.001.

**Table 3:** Patients’ scores in IES-R, BSHS-B, and AIS scales in both measurements. IES-R: Impact of Event Scale-Revised, BSHS-B: Burn Specific Health Scale-Brief, AIS: Athens Insomnia Scale

n=84	3 months		6 months		Change		P Wilcoxon signed-rank test
	Mean ± SD	Median (IQR)	Mean ± SD	Median (IQR)	Mean ± SD	Median (IQR)	
Avoidance	0.78±0.54	0.63 (0.38 ─ 1.13)	0.65±0.56)	0.5 (0.25 ─ 1)	-0.12±0.58)	-0.13 (-0.38 ─ 0.13)	0.038
Intrusion	1.35±0.71	1.25 (0.75 ─ 1.88)	1.33±0.64)	1.5 (0.81 ─ 1.75)	-0.02±0.77)	0 (-0.5 ─ 0.5)	0.820
Hyperarousal	0.63±0.51	0.5 (0.17 ─ 1)	0.61±0.46)	0.5 (0.17 ─ 0.83)	-0.02±0.6)	0 (-0.42 ─ 0.33)	0.650
Total IES-R	2.75±1.55	2.63 (1.42 ─ 3.88)	2.59±1.47)	2.6 (1.38 ─ 3.58)	-0.16±1.6)	-0.21 (-1.1 ─ 0.77)	0.246
Simple abilities	1.12±1.15	0.83 (0 ─ 1.67)	3.05±0.95	3.33 (2.67 ─ 3.67)	1.92±1.09	2 (1 ─ 2.67)	<0.001
Hand function	2.5±1.34	2.5 (1.7 ─ 3.6)	3.74±0.47	4 (3.7 ─ 4)	1.24±1.15	1 (0.2 ─ 2)	<0.001
Treatment regimens	2.17±1.06	2.3 (1.2 ─ 3)	2.95±1.05	3.4 (2.2 ─ 3.8)	0.78±1.13	0.8 (0 ─ 1.6)	<0.001
Heat sensitivity	2.56±1.02	2.8 (1.4 ─ 3.4)	2.63±0.91	2.9 (2.1 ─ 3.4)	0.07±1.26	0 (-0.8 ─ 1)	0.593
Work	1.56±1.23	1 (0.88 ─ 2.5)	2.44±1.02	2.5 (1.75 ─ 3.25)	0.88±1	0.75 (0.25 ─ 1.5)	<0.001
Affect	2.95±0.72	3 (2.43 ─ 3.43)	3.07±0.8	3.29 (2.57 ─ 3.71)	0.12±0.69	0 (-0.36 ─ 0.57)	0.133
Sexuality	2.42±1.16	2.67 (1.67 ─ 3.33)	2.6±1.08	2.67 (1.83 ─ 3.33)	0.18±1.11	0 (-0.67 ─ 1)	0.147
Interpersonal relationships	3.73±0.35	3.88 (3.5 ─ 4)	3.57±0.56	3.75 (3.25 ─ 4)	-0.16±0.55	0 (-0.5 ─ 0.25)	0.011
Body image	3.4±0.97	4 (3.25 ─ 4)	3.28±0.92	3.75 (2.75 ─ 4)	-0.11±0.93	0 (-0.5 ─ 0.25)	0.210
Total BSHS-B	102.15±25.79	104 (84 ─ 121.5)	122.21±24.82	126 (107 ─ 141.5)	20.06±21.55	18 (3.5 ─ 35)	<0.001
Insomnia scale (AIS)	8.32±4.69	8.5 (4 ─ 12)	5.92±3.9	6 (2 ─ 8)	-2.4±5.21	-2 (-5 ─ 1.5)	<0.001

At both time points, BSHS-B domains were significantly and negatively correlated with both IES-R and AIS scales (Table 4), indicating that greater PTSD and insomnia symptoms were significantly associated with poorer QoL. Conversely, a significant and positive correlation was found between PTSD and insomnia symptoms at both three and six months.

**Table 4:** Spearman’s correlation coefficients (rho) among patients’ scores in IES-R, BSHS-B, and AIS scales by measurement.

		3 months					6 months				
		Insomnia scale (AIS)	Avoidance	Intrusion	Hyperarousal	Total IES-R	Insomnia scale (AIS)	Avoidance	Intrusion	Hyperarousal	Total IES-R
Simple abilities	rho	-0.45	-0.29	-0.29	-0.34	-0.34	-0.63	-0.31	-0.46	-0.42	-0.46
	P	<0.001	0.006	0.006	0.002	0.002	<0.001	0.004	<0.001	<0.001	<0.001
Hand function	rho	-0.29	-0.25	-0.19	-0.21	-0.23	-0.35	-0.26	-0.34	-0.23	-0.33
	P	0.007	0.020	0.083	0.050	0.038	0.001	0.015	0.002	0.034	0.002
Treatment regimens	rho	-0.37	-0.49	-0.43	-0.39	-0.50	-0.45	-0.31	-0.35	-0.32	-0.35
	P	0.001	<0.001	<0.001	<0.001	<0.001	<0.001	0.004	0.001	0.003	0.001
Heat sensitivity	rho	-0.23	-0.34	-0.28	-0.25	-0.32	-0.55	-0.43	-0.55	-0.45	-0.55
	P	0.034	0.002	0.009	0.024	0.003	<0.001	<0.001	<0.001	<0.001	<0.001
Work	rho	-0.18	-0.28	-0.23	-0.25	-0.28	-0.44	-0.50	-0.40	-0.35	-0.46
	P	0.092	0.009	0.038	0.021	0.010	<0.001	<0.001	<0.001	0.001	<0.001
Affect	rho	-0.47	-0.63	-0.68	-0.51	-0.69	-0.61	-0.68	-0.67	-0.55	-0.71
	P	<0.001	<0.001	<0.001	<0.001	<0.001	<0.001	<0.001	<0.001	<0.001	<0.001
Sexuality	rho	-0.31	-0.50	-0.44	-0.29	-0.48	-0.57	-0.49	-0.50	-0.43	-0.53
	P	0.003	<0.001	<0.001	0.006	<0.001	<0.001	<0.001	<0.001	<0.001	<0.001
Interpersonal relationships	rho	-0.29	-0.49	-0.55	-0.46	-0.56	-0.32	-0.48	-0.34	-0.25	-0.39
	P	0.007	<0.001	<0.001	<0.001	<0.001	0.003	<0.001	0.002	0.023	<0.001
Body image	rho	-0.16	-0.52	-0.40	-0.22	-0.43	-0.25	-0.43	-0.32	-0.20	-0.34
	P	0.146	<0.001	<0.001	0.039	<0.001	0.023	<0.001	0.003	0.072	0.001
Total BSHS-B	rho	-0.44	-0.61	-0.55	-0.45	-0.60	-0.68	-0.60	-0.64	-0.53	-0.66
	P	<0.001	<0.001	<0.001	<0.001	<0.001	<0.001	<0.001	<0.001	<0.001	<0.001
Insomnia scale (AIS)	rho	1.00	0.28	0.35	0.56	0.45	1.00	0.51	0.67	0.61	0.68
	P		0.010	0.001	<0.001	<0.001		<0.001	<0.001	<0.001	<0.001

Via multiple linear regression, it was found that at three months, a greater extent of burn was significantly associated with worse QoL regarding simple abilities (β = -0.006; p = 0.001), hand function (β = -0.006; p = 0.001), and affect (β = -0.001; p = 0.009); Table 5. Also, greater PTSD symptoms were significantly associated with worse QoL regarding simple abilities (β = -0.042; p = 0.005), treatment regimens (β = -0.030; p = 0.002), affect (β = -0.034; p < 0.001), sexuality (β = -0.045; p < 0.001), interpersonal relationships (β = -0.011; p < 0.001), body image (β = -0.033; p < 0.001), and worse overall QoL (β = -0.042; p < 0.001). Females had significantly worse QoL regarding hand function (β = -0.095; p = 0.042) and body image (β = -0.087; p = 0.002). Conversely, greater age was significantly associated with better QoL regarding work (β = 0.003; p = 0.006), sexuality (β = 0.003; p < 0.001), and body image (β = 0.002; p = 0.007). Having complications was significantly associated with worse QoL regarding treatment regimens (β = -0.061; p = 0.029), affect (β = -0.030; p = 0.022), and interpersonal relationships (β = -0.022; p = 0.001). Suffering from insomnia was significantly associated with worse QoL regarding treatment regimens (β = -0.071; p = 0.028) and heat sensitivity (β = -0.071; p = 0.023). Moreover, greater duration of hospitalization was significantly associated with worse QoL regarding heat sensitivity (β = -0.001; p = 0.022), work (β = -0.002; p = 0.004), and worse overall QoL (β = -0.001; p = 0.001). Also, patients who lived alone had significantly worse QoL regarding affect (β = -0.041; p = 0.004) and interpersonal relationships (β = -0.019; p = 0.006). Having an autograft transplantation was significantly associated with worse QoL regarding treatment regimens (β = -0.112; p < 0.001), having a superficial-partial burn injury was significantly associated with better QoL regarding heat sensitivity (β = 0.125; p < 0.001), and having a superficial burn injury was significantly associated with better QoL regarding sexuality (β = 0.073; p = 0.021).

**Table 5:** Multiple linear regression analyses results with BSHS-B subscales at three months as dependent variables, in a stepwise method. Note. The dependent variables were logarithmically transformed prior to the analysis due to lack of normality in their distribution; +regression coefficient; ++Standard Error

Dependent variable:	Independent variables:	β+	SE++	P
Simple abilities	Extent of burn (%)	-0.006	0.002	0.001
	Total IES-R	-0.042	0.015	0.005
Hand function	Extent of burn (%)	-0.006	0.002	0.001
	Gender (Females vs Males)	-0.095	0.046	0.042
Treatment regimens	Total IES-R	-0.030	0.010	0.002
	Autograft Transplantation (yes vs no)	-0.112	0.027	<0.001
	Insomnia (yes vs no)	-0.071	0.032	0.028
	Complications (yes vs no)	-0.061	0.027	0.029
Heat sensitivity	Superficial partial burn (yes vs no)	0.125	0.030	<0.001
	Duration of hospitalization	-0.001	0.001	0.022
	Insomnia (yes vs no)	-0.071	0.030	0.023
Work	Duration of hospitalization	-0.002	0.001	0.004
	Age	0.003	0.001	0.006
Affect	Total IES-R	-0.034	0.004	<0.001
	Extent of burn (%)	-0.001	0.001	0.009
	Living alone (yes vs no)	-0.041	0.014	0.004
	Complications (yes vs no)	-0.030	0.013	0.022
Sexuality	Total IES-R	-0.045	0.010	<0.001
	Age	0.003	0.001	<0.001
	Superficial (yes vs no)	0.073	0.031	0.021
Interpersonal relationships	Total IES-R	-0.011	0.002	<0.001
	Complications (yes vs no)	-0.022	0.006	0.001
	Living alone (yes vs no)	-0.019	0.007	0.006
Body image	Total IES-R	-0.033	0.008	<0.001
	Gender (Females vs Males)	-0.087	0.027	0.002
	Age	0.002	0.001	0.007
Total BSHS-B	Total IES-R	-0.042	0.007	<0.001
	Duration of hospitalization	-0.001	0.000	0.001

At six months, it was found, via multiple linear regression, that a greater extent of burn was significantly associated with worse QoL regarding hand function (β = -0.001; p = 0.006), treatment regimens (β = -0.005; p < 0.001), and worse overall QoL (β = -0.003; p < 0.001); Table 6. Furthermore, greater PTSD symptoms were significantly associated with worse QoL regarding hand function (β = -0.007; p = 0.049), treatment regimens (β = -0.029; p = 0.001), heat sensitivity (β = -0.029; p = 0.004), work (β = -0.038; p < 0.001), affect (β = -0.040; p < 0.001), sexuality (β = -0.038; p = 0.001), interpersonal relationships (β = -0.016; p < 0.001), body image (β = -0.025; p = 0.001), and worse overall QoL (β = -0.040; p < 0.001). Females had significantly worse QoL regarding body image (β = -0.061; p = 0.008). On the contrary, greater age was significantly associated with better QoL regarding sexuality (β = 0.003; p < 0.001) and body image (β = 0.002; p = 0.002). Suffering from insomnia was significantly associated with worse QoL regarding simple abilities (β = -0.098; p < 0.001), heat sensitivity (β = -0.059; p = 0.045), and sexuality (β = -0.072; p = 0.032). Moreover, greater duration of hospitalization was significantly associated with worse QoL regarding work (β = -0.002; p = 0.002), affect (β = -0.001; p = 0.001), and body image (β = -0.001; p = 0.006). A greater educational level was significantly associated with better QoL regarding simple abilities (β = 0.028; p = 0.029) and hand function (β = 0.010; p = 0.034). Also, patients who lived alone had significantly worse QoL regarding affect (β = -0.045; p = 0.008), and those with non-Greek nationality had significantly worse QoL regarding heat sensitivity (β = -0.090; p = 0.009). A greater ABSI score was significantly associated with worse QoL regarding simple abilities (β = -0.019; p = 0.004). Having an autograft transplantation was significantly associated with worse QoL regarding work (β = -0.089; p = 0.001), and having a thermal burn was significantly associated with worse QoL regarding heat sensitivity (β = -0.092; p = 0.013) and sexuality (β = -0.091; p = 0.036). Moreover, having an escharotomy was significantly associated with better QoL regarding hand function (β = 0.041; p = 0.001), while having an inhalation injury was significantly associated with worse QoL regarding interpersonal relationships (β = -0.083; p < 0.001) and sexuality (β = -0.093; p = 0.028).

**Table 6:** Multiple linear regression analyses results with BSHS-B subscales at six months as dependent variables, in a stepwise method. Note. The dependent variables were logarithmically transformed prior to the analysis due to lack of normality in their distribution; +regression coefficient; ++Standard Error

Dependent variable:	Independent variables:	β+	SE++	P
Simple abilities	Insomnia (yes vs. no)	-0.098	0.026	<0.001
	ABSI Score	-0.019	0.006	0.004
	Educational level	0.028	0.013	0.029
Hand function	Total IES-R	-0.007	0.003	0.049
	Educational level	0.010	0.005	0.034
	Escharotomy (yes vs. no)	0.041	0.013	0.001
	Extent of burn (%)	-0.001	0.000	0.006
Treatment regimens	Extent of burn (%)	-0.005	0.001	<0.001
	Total IES-R	-0.029	0.008	0.001
Heat sensitivity	Total IES-R	-0.029	0.010	0.004
	Nationality (Other vs. Greek)	-0.090	0.034	0.009
	Thermal Burn (yes vs. no)	-0.092	0.036	0.013
	Insomnia (yes vs. no)	-0.059	0.029	0.045
Work	Total IES-R	-0.038	0.009	<0.001
	Autograft Transplantation (yes vs. no)	-0.089	0.027	0.001
	Duration of hospitalization	-0.002	0.000	0.002
Affect	Total IES-R	-0.040	0.005	<0.001
	Duration of hospitalization	-0.001	0.000	0.001
	Living alone (yes vs. no)	-0.045	0.016	0.008
Sexuality	Total IES-R	-0.038	0.011	0.001
	Age	0.003	0.001	<0.001
	Thermal Burn (yes vs. no)	-0.091	0.043	0.036
	Presence of inhalation injury (yes vs. no)	-0.093	0.042	0.028
	Insomnia (yes vs. no)	-0.072	0.033	0.032
Interpersonal relationships	Presence of inhalation injury (yes vs. no)	-0.083	0.019	<0.001
	Total IES-R	-0.016	0.004	<0.001
Body image	Total IES-R	-0.025	0.007	0.001
	Duration of hospitalization	-0.001	0.000	0.006
	Age	0.002	0.001	0.002
	Gender (Females vs. Males)	-0.061	0.022	0.008
Total BSHS-B	Total IES-R	-0.040	0.005	<0.001
	Extent of burn (%)	-0.003	0.001	<0.001

## Discussion

This study aimed to evaluate QoL, PTSD, and insomnia in patients with burn injuries and to explore the interrelationships among these variables. Assessments were conducted at three and six months following hospital discharge to capture the temporal trajectory of psychological well-being and QoL.

In this study, participants demonstrated mild-to-moderate PTSD symptomatology at both three- and six-month post-discharge. Although the reduction did not reach statistical significance, mean scores at both timepoints exceeded the clinical threshold of 1.5-1.6, indicating the persistence of clinically relevant post-traumatic stress symptoms. These findings align with previous research utilizing the IES-R index in burn survivors. Notably, de Vries et al. [18], using a variant of the IES-R (IESplus), reported persistent PTSD symptoms at three months post-injury (mean total IESplus score 21.5, SD = 23.9) and six months (mean = 20.6, SD = 23.2), particularly in the Avoidance (from 8.4, SD = 9.3, to 8.0, SD = 9.1) and Hyperarousal domains (from 5.3, SD = 6.1, to 4.9, SD = 5.9). Our findings further demonstrated a statistically significant reduction in Avoidance domain scores from three to six months (from 0.78 to 0.65, p = 0.038), whereas intrusion and hyperarousal domain scores remained unchanged. This selective decline in the avoidance domain aligns with findings by Fauerbach et al. [19], who observed that behavioral avoidance symptoms often improve more rapidly due to patients’ reintegration into daily routines and the development of adaptive coping strategies. Similarly, Sveen et al. [20] validated the IES-R's sensitivity to change in burn survivors and highlighted that scores exceeding 1.6 signal clinically significant distress, even in the absence of a formal PTSD diagnosis.

Quality of life improved significantly between three and six months post-discharge, as measured by the BSHS-B. Specifically, the Simple abilities domain increased from 1.12 to 3.05, the Hand function domain from 2.50 to 3.74, the Treatment regimens domain from 2.17 to 2.95, and the Work domain from 1.56 to 2.44. In contrast, the interpersonal relationships domain slightly declined from 3.73 to 3.57, while the total BSHS-B score increased by 20.06 points (SD = 21.55). These results are consistent with those reported by Willebrand et al. [21], who observed significant improvement in function and skin involvement domains at six months. Additional studies conducted in Nepal and Spain identified substantial clinical gains in the simple abilities and hand function domains [22]. Johnson et al. [23] also reported declines in the interpersonal relationships domain (≈ 0.2-0.3 points) between three and six months, paralleling our findings (3.73 → 3.57).

Despite the established utility of the AIS in various clinical populations, including individuals with chronic diseases, psychiatric disorders, and healthcare workers, no studies were identified that have applied the AIS specifically to burn survivors. Previous research has often relied on alternative tools such as the Insomnia Severity Index (ISI) or the Pittsburgh Sleep Quality Index (PSQI), which assess broader constructs of sleep quality rather than clinical insomnia per se. For instance, Lee et al. [24] used a single sleep satisfaction item from the Young Adult Burn Outcome Questionnaire (YABOQ) and found that nearly half of the participants remained dissatisfied with their sleep at six months, particularly those with extensive TBSA involvement. Liu et al. [25], employing the PSQI, reported a mean score of 9.4 (SD = 4.1) in patients with hypertrophic scarring. However, this study lacked longitudinal assessment. The present study addresses this gap and highlights the relevance of standardized instruments such as the AIS in assessing insomnia post-burn.

A strong negative correlation was observed between total BSHS-B and IES-R scores at both timepoints (rho = -0.60 to -0.66), confirming that greater psychological distress is associated with reduced QoL in burn survivors. This is consistent with the study by Ricci et al. [26], where 73 Brazilian patients had a mean total BSHS-B score of 128.1 (SD = 18.9), with a significant negative correlation with the IES scale (-0.55), while the means for the Intrusion and Avoidance subscales were relatively low (Intrusion mean = 23.8, SD = 21.4, Avoidance mean = 38.4, SD = 19.1). Likewise, Berg et al. [27], in their study of 146 burn patients, recorded an IES-R subscale Intrusion mean of 8.2 (SD = 9.0), an Avoidance mean of 7.5 (SD = 8.9), and a Hyperarousal mean of 7.0 (SD = 9.3), with no statistically significant differences between time groups, indicating a stable presence of psychological symptoms up to 49 months after the burn. The present findings support a biopsychosocial model of burn recovery and underscore the necessity of integrated care approaches.

Notably, our study is among the first to examine the association between QoL and insomnia using the BSHS-B and AIS in tandem. The observed significant negative correlation suggests that insomnia may exert a broader detrimental impact on QoL than PTSD alone. This underscores the need for routine sleep assessments during burn rehabilitation.

Regression analyses at three months revealed that greater burn extent was inversely associated with simple abilities, hand function, and affect domains-findings corroborated by Tibebu et al. [28], who noted that TBSA ≥20% predicted worse QoL. However, a systematic review by Spronk et al. [29] found TBSA to be an inconsistent predictor, with significant associations reported in only 29% of studies. Conversely, the same review confirms that indicators such as length of stay and number of procedures possess stronger predictive value, which is supported by the present study, where longer length of stay negatively impacted the heat sensitivity, work, and overall QoL domains. PTSD severity significantly impacted multiple QoL domains, a trend similarly reported by Spronk et al. [29], who identified PTSD symptoms among the top five predictors of poor post-burn QoL. Additionally, Druery et al. [30] found that female gender and prior psychiatric diagnoses adversely affected the affect and body image domains-findings echoed in our results, where female gender negatively influenced hand function and body image. The presence of complications such as amputation or grafting was also associated with reduced scores in the treatment regimens, affect, and interpersonal relationships domains, consistent with data by Tibebu et al. [28], where patients with amputation had increased odds of poor QoL (AOR = 2.48, 95% CI: 1.33-4.63, p = 0.004). Similarly, the presence of comorbidities was associated with significantly lower QoL (AOR = 3.73, p < 0.001), confirming the importance of combined physical and psychological burdens as predictors. Finally, findings of the present study, not extensively reported elsewhere, include the negative impact of insomnia on the heat sensitivity and treatment regimens subscales, as well as the positive association of age with work, sexuality, and body image subscales, which merit further exploration in future multivariate predictive studies.

At six months post-injury, multivariate analyses confirmed that a greater extent of burned surface area, increased PTSD symptoms, insomnia, prolonged hospitalization, and female gender remained significant negative predictors of overall QoL and its subdomains. These findings align with those by Spronk et al. [29], Tibebu et al. [28], and Druery et al. [30], who, although focusing on 12-month outcomes, reported similar associations between PTSD symptoms and diminished affect and body image domains.

Limitations of the study

Several limitations should be acknowledged. First, the design precludes causal inferences and limits the ability to assess dynamic changes over longer periods. Although follow-up was conducted at two timepoints, the duration was limited to six months, restricting insight into long-term outcomes.

Second, the relatively small sample size reflects the inherent recruitment challenges in burn populations and may affect generalizability. Additionally, the use of convenience sampling from two hospitals in Attica may not represent the broader population of burn survivors across Greece.

Third, although the use of standardized tools such as the BSHS-B, IES-R, and AIS strengthens internal validity, the lack of comparable studies using identical instruments restricts external benchmarking.

Future research should incorporate larger, multicenter cohorts with extended follow-up periods beyond 12 months to better evaluate causal relationships and the efficacy of targeted interventions. Moreover, longitudinal designs that integrate sleep and psychological assessments into holistic rehabilitation programs are warranted.

Confounding factors

Potential confounding factors in this study include pain intensity, anatomical site of injury, burn severity (TBSA), early rehabilitation, and duration of hospitalization. These variables may influence both psychological outcomes and quality of life in burn survivors. Although not systematically recorded in the present study, their potential impact is acknowledged, and future research should consider their inclusion to better understand recovery trajectories.

## Conclusions

This study highlights the multifaceted impact of burn injuries, including physical trauma, psychological distress, and sleep disturbances. While quality of life improved within six months post-discharge, symptoms of PTSD and insomnia persisted, with insomnia, assessed for the first time in a Greek burn-injured population via the Athens Insomnia Scale, emerging as a significant independent predictor of post-burn QoL. Greater burn extent, prolonged hospitalization, complications, and female sex were also identified as consistent negative predictors of both overall and domain-specific BSHS-B outcomes.

These findings emphasize the importance of a comprehensive biopsychosocial approach to burn rehabilitation, incorporating routine assessment and management of mental health and sleep problems. Future research should focus on large-scale, longitudinal studies across diverse populations to further clarify these relationships and support the development of evidence-based interventions.
